# Advancing stroke therapy: A deep dive into early phase of ischemic stroke and recanalization

**DOI:** 10.1111/cns.14634

**Published:** 2024-02-20

**Authors:** Qianyan He, Yueqing Wang, Cheng Fang, Ziying Feng, Meifang Yin, Juyang Huang, Yinzhong Ma, Zhizhun Mo

**Affiliations:** ^1^ Department of Neurology, Stroke Center The First Hospital of Jilin University Jilin China; ^2^ Institute of Biomedicine and Biotechnology Shenzhen Institute of Advanced Technology, Chinese Academy of Sciences Shenzhen Guangdong China; ^3^ School of Pharmaceutical Sciences (Shenzhen) Sun Yat‐sen University Shenzhen Guangdong China; ^4^ Emergency Department, Shenzhen Traditional Chinese Medicine Hospital The Fourth Clinical Medical College of Guangzhou University of Chinese Medicine Shenzhen Guangdong China

**Keywords:** acute ischemic stroke, blood–brain barrier, ischemia–reperfusion injury, neutrophil

## Abstract

Ischemic stroke, accounting for the majority of stroke events, significantly contributes to global morbidity and mortality. Vascular recanalization therapies, namely intravenous thrombolysis and mechanical thrombectomy, have emerged as critical interventions, yet their success hinges on timely application and patient‐specific factors. This review focuses on the early phase pathophysiological mechanisms of ischemic stroke and the nuances of recanalization. It highlights the dual role of neutrophils in tissue damage and repair, and the critical involvement of the blood–brain barrier (BBB) in stroke outcomes. Special emphasis is placed on ischemia–reperfusion injury, characterized by oxidative stress, inflammation, and endothelial dysfunction, which paradoxically exacerbates cerebral damage post‐revascularization. The review also explores the potential of targeting molecular pathways involved in BBB integrity and inflammation to enhance the efficacy of recanalization therapies. By synthesizing current research, this paper aims to provide insights into optimizing treatment protocols and developing adjuvant neuroprotective strategies, thereby advancing stroke therapy and improving patient outcomes.

## Graphical abstract

Visualizing the path to stroke recovery: This graphic abstract illuminates key insights into the early phase of ischemic stroke, emphasizing the groundbreaking recanalization therapies that are reshaping patient care. By illustrating the intricate dance of neutrophils, which play a dual role in tissue damage and repair, and shedding light on the critical importance of maintaining blood–brain barrier integrity, this depiction offers a clear view of the complex pathophysiology underlying stroke. Furthermore, it highlights the paradoxical effects of ischemia‐reperfusion injury, suggesting novel molecular targets for enhancing recanalization therapy efficacy. Our review charts a course toward optimizing treatment strategies, underpinning the development of next‐generation neuroprotective approaches in the quest to improve outcomes for stroke patients.

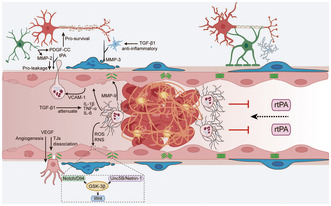

## 1 INTRODUCTION

Stroke is the leading cause of death and long‐term disability which remains a significant global burden, with an estimated annual incidence of 13 million cases worldwide.^1^, ^2^, ^3^ Ischemic stroke, accounting for approximately 80% of stroke cases, is characterized by the interruption of blood flow to the brain due to arterial occlusion. Vascular recanalization therapy, the foremost approach in the management of cerebral ischemia, aims to restore blood flow by reopening occluded blood vessels which supply the affected area and has shown considerable promise in treating cerebral ischemia by salvaging brain ischemic penumbra.^4^, ^5^, ^6^ The timely and successful implementation of recanalization techniques has been associated with improved neurological recovery and reduced disability. Nevertheless, challenges and controversies persist in this field. The expeditious administration of ischemia–reperfusion therapy is constrained by a critical temporal parameter. Intravenous thrombolysis is necessitated to be administered within a 4.5‐h period post‐onset, while mechanical thrombectomy may be contemplated for up to 24 h from onset in patients who satisfy the criteria as delineated by imaging evidence and in accordance with clinical guideline directives.^7^ Although the reperfusion phase is essential for salvaging viable neurons, it paradoxically initiates ischemia–reperfusion injury, a major concern that precipitates a cascade of deleterious pathophysiological events. These events include oxidative stress, calcium overload, inflammation, and endothelial cell dysfunction, each contributing to the complex interplay that exacerbates cerebral damage rather than mitigating it.^7^, ^8^, ^9^, ^10^ The blood–brain barrier (BBB) is a highly selective structural and functional barrier between the blood and brain tissue, creating a unique microenvironment required for regular brain function and homeostasis. Disruption of the BBB is a hallmark of cerebral ischemia–reperfusion injury, often leading to deadly complications like hemorrhagic transformation and aggravating neuroinflammation after stroke.^11^ Understanding the nuances of this multifaceted secondary injury cascade is key to developing optimized recanalization therapies and adjuvant neuroprotective agents for ischemic stroke.

Through ongoing research efforts, the field of acute cerebral ischemia–reperfusion injury is advancing, uncovering new insights into its underlying mechanisms, and identifying potential therapeutic avenues. This review focuses on synthesizing current knowledge on the pathophysiological mechanisms in the early phase following ischemic stroke and vascular recanalization. The initial neutrophil response, release of proteases from multiple sources, endothelial programmed cell death induction, and signaling pathways maintaining BBB integrity represent intricately connected processes elucidated by ongoing research. In particular, neutrophil‐mediated oxidative and proteolytic damage, modulation of cell death pathways, cytokine and chemokine signaling axes, and pharmacological agents stabilizing mediators like β‐catenin emerge as key areas. Targeting these molecular contributors and optimizing treatment protocols to leverage endogenous repair mechanisms holds promise for enhancing the efficacy of recanalization therapies. A deeper understanding of the acute injury network will aid the development of adjuvant drugs and approaches to counteract the early damage cascade, improving prognosis after ischemic stroke.

## 2 THE MECHANISM OF EARLY NEUTROPHIL ACTIVATION ON CEREBROVASCULAR INJURY

Neutrophils, as key players in the innate immune system, fulfill a crucial role in the pathogenesis of stroke. These abundant leukocytes, constituting approximately 50–70% of the total white blood cell population, are rapidly mobilized to sites of brain ischemia, in response to danger signals.^12^ Neutrophils possess remarkable capabilities for rapid activation and the release of pro‐inflammatory factors, enabling them to mount a swift inflammatory response.^13^ This section will delve into the role of neutrophils in stroke disease from the perspective of the innate immune system, highlighting their significant presence in the blood and the mechanisms underlying their rapid inflammatory response.

**FIGURE 1:**
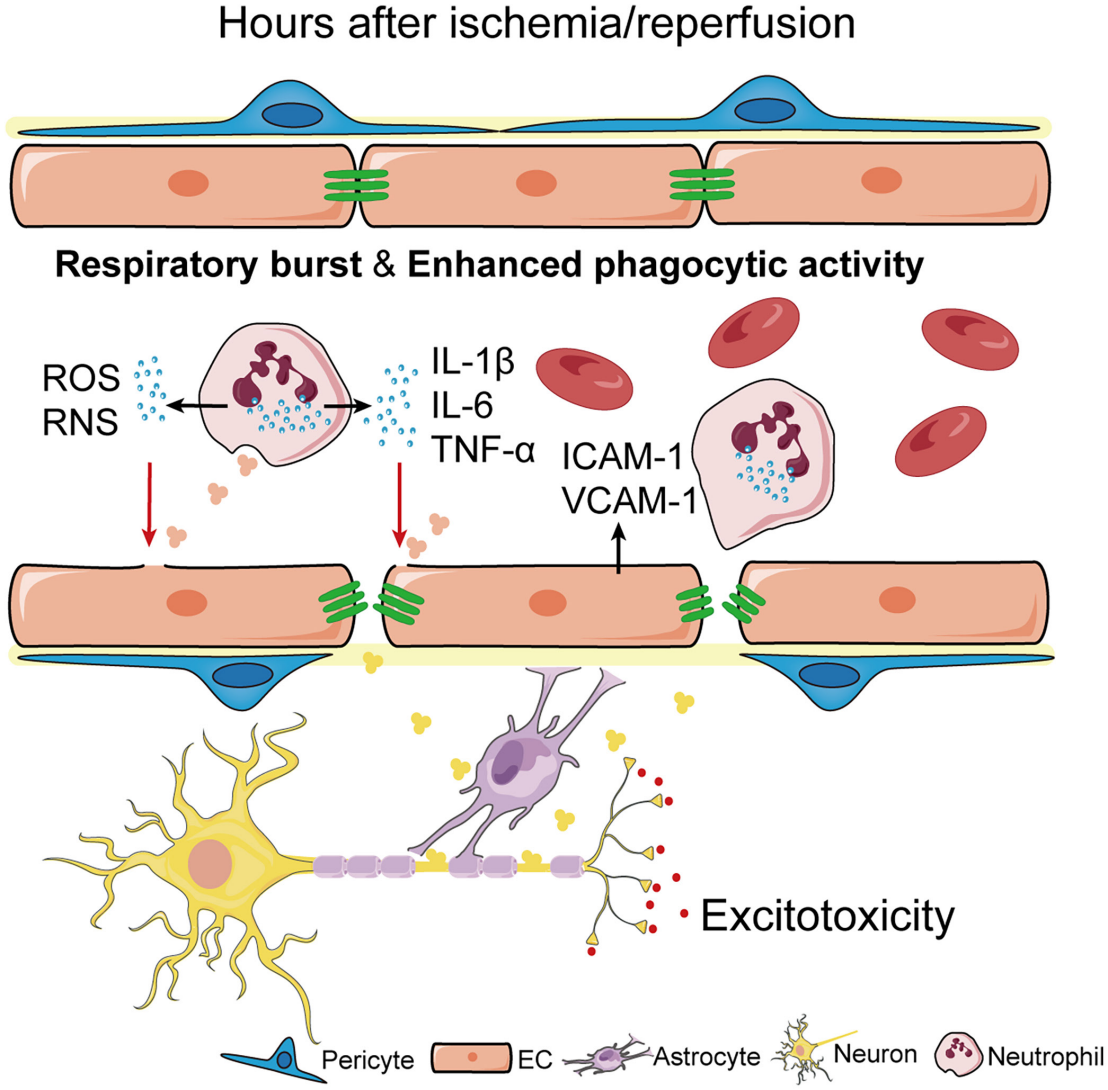
Neutrophil activation and inflammatory cascade post‐reperfusion. This figure depicts the cascade of events following ischemia/reperfusion, starting with excitotoxicity‐induced neuronal death leading to the accumulation of cell debris and Damage‐Associated Molecular Patterns (DAMPs). This process aids in clearing damaged cells through enhanced phagocytic activity but may also intensify inflammation by degranulation. Concurrently, pro‐inflammatory factors activate endothelial cells, prompting the upregulation of adhesion molecules like ICAM‐1 and VCAM‐1, which facilitate leukocyte adhesion and migration. Furthermore, activated neutrophils undergo a respiratory burst, producing an influx of ROS and RNS. These reactive species further activate the programmed death of endothelial cells and surrounding tissue, perpetuating the cycle of inflammation and immune cell recruitment.

The functions of neutrophils can be broadly categorized into three major areas: phagocytosis, respiratory burst, and the formation of neutrophil extracellular traps (NETs). Activated neutrophils exhibit enhanced phagocytic activity, engulfing cellular debris during acute ischemic injury (Figure 1). This can promote clearance of damaged cells but may also exacerbate inflammation. Upon activation, neutrophils release inflammatory factors, such as interleukin‐1β (IL‐1β), tumor necrosis factor‐alpha (TNF‐α), and interleukin‐6 (IL‐6), which propagate the inflammatory response.^14^ The early release of inflammatory factors further activates endothelial cells, leading to the upregulation of adhesion molecules including intercellular adhesion molecule‐1 (ICAM‐1) and vascular cell adhesion molecule‐1 (VCAM‐1), which facilitate leukocyte adhesion and migration. Downregulate the expression of proinflammatory cytokines, and adhesion molecules could alleviate BBB disruption and improve neurological deficits.^15^, ^16^ The aforementioned research collectively demonstrates that inflammatory factor‐mediated activation of endothelial cells occurs swiftly subsequent to cerebral ischemia and reperfusion events.

In addition to inflammatory mediators, activated neutrophils also undergo respiratory burst, rapidly generating reactive oxygen species (ROS) and reactive nitrogen species (RNS) such as peroxynitrite that induce oxidative damage to cerebrovascular endothelial cells.^11^, ^17^ Neutrophil ROS and RNS production are early contributors to cerebrovascular damage after ischemic stroke as demonstrated in Figure 1.

**FIGURE 2:**
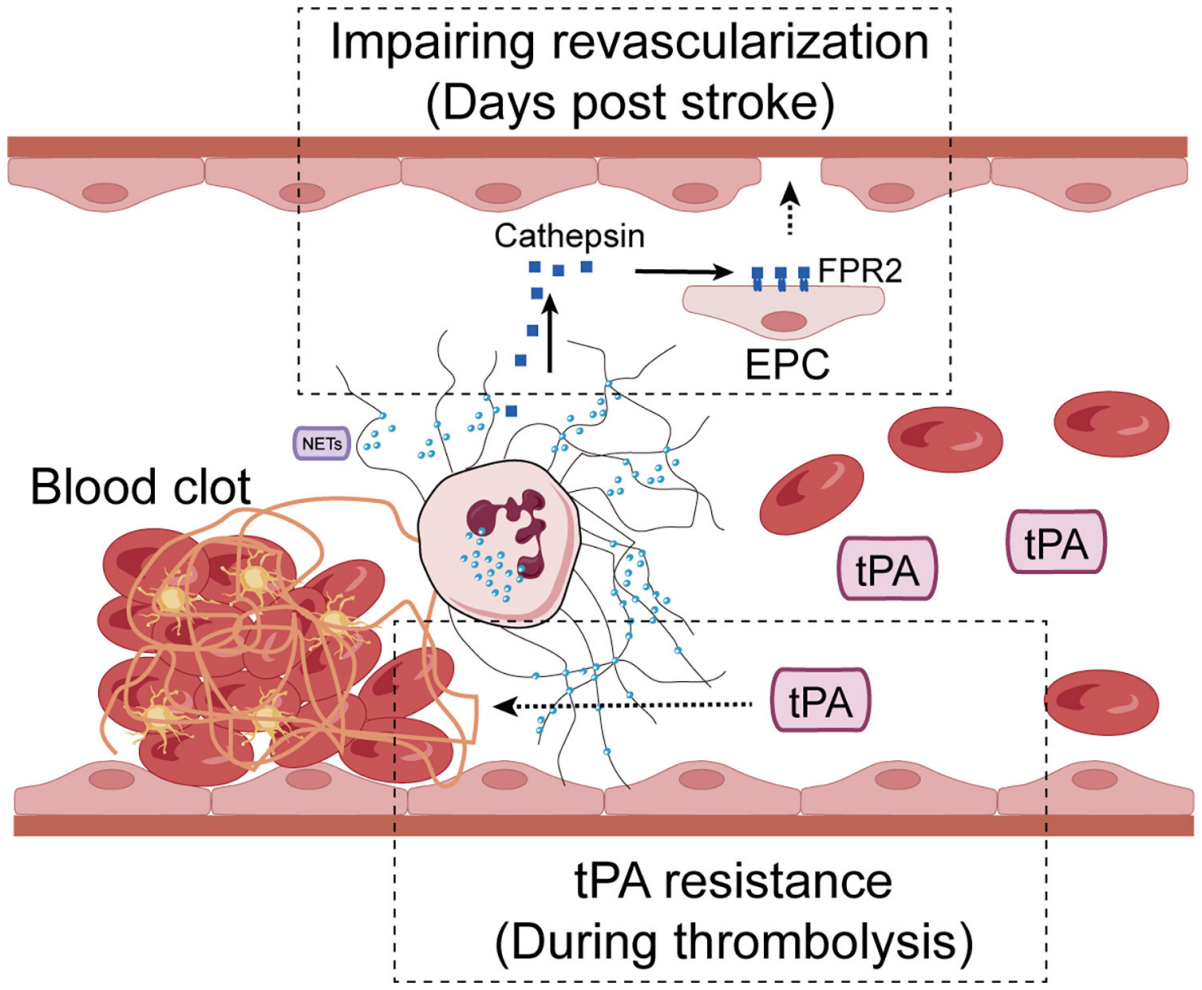
Dual impact of NETs following stroke. Following a stroke, the formation of NETs emerges as a key factor influencing both vascular recovery and thrombolytic therapy. In the upper segment, NETs are shown to hinder the process of revascularization, a critical step in post‐stroke healing. Studies have highlighted how NET components, including decondensed chromatin and granular proteins such as cathepsin, disrupt endothelial cell function and angiogenesis, essential for restoring blood flow to ischemic brain tissue. This interference with new vessel formation signifies a substantial obstacle in tissue repair and functional recovery. The lower part of the illustration delves into the interaction between NETs and rtPA. NETs are portrayed as barriers to effective thrombolysis. Research indicates that the dense meshwork of NETs can physically impede rtPA access to clots, thereby reducing its efficacy. Additionally, the presence of NETs has been linked to increased clot stability, posing a challenge to rtPA‐mediated clot breakdown. Thus, this figure encapsulates the dual, albeit counterproductive, roles of NETs in post‐stroke pathology, demonstrating their defensive nature in immune response while also revealing their potential to complicate recovery and treatment.

Finally, neutrophil activation triggers NETs formation, releasing web‐like chromatin structures decorated with proteases, including matrix metalloproteinases (MMPs), elastase, and cathepsins, which degrade extracellular matrix components and contribute to tissue remodeling. In a rat thromboembolic stroke model, Justicia et al. found increased neutrophil infiltration and MMP‐9 level in the ischemic hemisphere within hours of reperfusion.^18^ The role of NETs in stroke and thrombolytic therapy has been the focus of recent research (Figure 2). It was found that the release of NETs impairs cerebrovascular integrity and exacerbates tPA‐induced intracerebral hemorrhage, which suggests that NETs significantly contribute to tPA‐induced blood–brain barrier breakdown in the ischemic brain.^19^ More importantly, pharmacological modulation of NETs can reverse tPA resistance. Researchers have found that administration of DNase‐I, which promotes NETs lysis, recanalizes the occluded vessel improving photothrombotic stroke outcome.^20^ This suggests that targeting NETs may ameliorate thrombolytic therapy for ischemic stroke by reducing tPA‐associated hemorrhage. It was worth noticing that the production of NETs from neutrophils did not peak at hours post‐recanalization, but 3–5 days post‐stroke. The depletion of neutrophils effectively reduced the formation of NETs in mice subjected to traumatic brain injury. Furthermore, neutrophils accumulate in the ischemic cortex during the progression of ischemic stroke and release of NETs impairs vascular remodeling during stroke recovery. The degradation of NETs or inhibition of NETs' formation significantly enhanced neovascularization at 14 days post‐stroke.^21^ These findings suggest that NETs not only contribute to the acute phase of stroke but also influence the chronic recovery period. By impairing revascularization and vascular remodeling, NETs could potentially hinder the brain's natural healing process after stroke. Therefore, strategies aimed at modulating NETs' formation and function in the acute stage post‐stroke could also be beneficial for improving long‐term recovery after stroke.

In summary, the collective effects of neutrophil activation result in extensive cerebrovascular injury in the context of cerebral ischemia–reperfusion. In the initial period after reperfusion, neutrophil‐mediated oxidative damage, protease release, and NETs are key factors underlying endothelial cell injury and microvascular disruption. Targeting these early neutrophil‐dependent processes represents a promising therapeutic strategy for mitigating cerebrovascular injury after ischemic stroke.

## 3 CELLULAR SOURCES AND DYNAMICS OF MMPs RELEASE

MMPs are a group of enzymes involved in the degradation and remodeling of the extracellular matrix. In the context of cerebral ischemia–reperfusion injury, MMPs are upregulated in brain tissue and blood, which play a significant role in mediating the pathophysiological processes associated with hemorrhagic transformation and intracerebral hemorrhage following vascular recanalization therapy.^22^, ^23^, ^24^ Various cell types, including neutrophils, monocytes, and macrophages, as well as cells of neurovascular unit, contribute to the production of MMPs in the ischemic brain (Table 1).^22^, ^25^, ^26^, ^27^ Understanding the source of MMPs will help further elucidate the mechanism of their action on the deterioration of the BBB.

**TABLE 1:** Different cellular sources of MMPs.

MMPs	Cellular sources	Reference
MMP‐2	Endothelial cell, Astrocyte	22, 28
MMP‐3	Neuron, Endothelial cell, Microphage, Pericyte, Macrophage	43, 44, 45
MMP‐8	Neutrophils	^22^
MMP‐9	Neutrophils, Endothelial cell, Macrophage	24, 31, 40, 41
MMP‐10	Neuron	22, 27
MMP‐13	Neuron	^26^
MMP‐14	Endothelial cell, Astrocyte	22, 28

In the pathophysiology of ischemic stroke, the initial response among the MMPs is predominantly observed in MMP‐2. This is likely attributed to its usual presence in an inactive, latent form, which can be rapidly converted into an active state. Astrocytes are identified as the primary cellular sources of MMP‐2, typically secreting it from their end feet, where it exerts effects on adjacent structures. The activation process of MMP‐2 involves with the participation of MMP‐14, located at the astrocytic end‐foot interfacing with endothelial cells.^28^ An early rise within 2 hours after stroke of active MMP‐2 degrades type IV collagen and laminin in the vascular basement membrane and leads to the degradation of type IV collagen and laminin in the vascular basement membrane. This degradation is a precursor to the disruption of the BBB and subsequent hemorrhagic transformation.^29^, ^30^

MMP‐9 is critically involved in the initial phase of cerebral ischemia–reperfusion injury, exhibiting significant influence within the initial 24‐hour period. Serving as pivotal elements of the innate immune response, neutrophils are identified as the primary cellular contributors to MMP‐9 production. Upon activation, neutrophils release MMP‐9, which can degrade the basement membrane, leading to increased vascular permeability and the disruption of tight junctions.^24^, ^31^ Inhibiting neutrophil infiltration has been shown to diminish MMP‐9 concentrations in the brain within the first24 hours after stroke.^18^In rodent MCAO models, plasma activity of MMP‐9 and MMP‐2 is increased within 3 to 8 hours of stroke onset. In primate models of stroke, a transient increase in plasma MMP‐9 activity is detected within 2 hours after reperfusion.^32^, ^33^, ^34^, ^35^ Additionally, in humans, MMP‐9 mRNA levels in peripheral blood leukocytes experience an elevation at 3–5 hours post‐stroke, returning to baseline after 24 h.^36^, ^37^ Blood replacement therapy significantly reduces infarctions, improves neurological deficits, and substantially decreases neutrophils in peripheral blood in a murine transient MCAO model. It also reduced the cytokine storm in plasma and decreased levels of MMP‐9 in the plasma and brain at different time points post‐stroke. Interestingly, the addition of MMP‐9 to the blood diminished the protective effect of the blood replacement therapy.^38^ Researchers found that early BBB breakdown is prevented in chimeric mice with leukocytes deficient in MMP‐9 while disruption of BBB did occur in chimeric mice with intact MMP‐9 in leukocytes.^39^ Evidence above suggested that MMP‐9 mainly released by leukocytes (mainly by neutrophils) may contribute to early breakdown of the BBB and early hemorrhagic transformation (HT). Similarly, monocytes and macrophages infiltrating the ischemic brain and endothelial cells also contribute to MMP‐9 production.^40^, ^41^ However, MMP‐9 also facilitates positive neurovascular remodeling at later time points,^42^ highlighting its complex roles at different phases of injury and recovery.

After cerebral ischemia/reperfusion, MMP‐3 was activated and observed in neurons, endothelial cells, and macrophages and participated in the pathogenesis of stroke.^43^, ^44^ The activation of MMP‐9 requires the engagement of active form of MMP‐3. In thrombotic MCAO with tPA mice, both the mRNA and protein level of MMP‐3 were increased in capillary endothelium.^45^ While other members of the MMP family are recognized for their association with early BBB disruption following cerebral ischemia, their specific roles in HT and their impact on neurological outcomes remain less elucidated and require further investigation.

In summary, various cellular sources contribute to the production of MMPs in cerebral ischemia–reperfusion injury. Neutrophils, monocytes, and macrophages, and cells of neurovascular unit all play a role in MMP secretion, leading to the degradation of the basement membrane, tight junctions, and extracellular matrix components. These events increase the risk of hemorrhagic transformation and intracerebral hemorrhage following vascular recanalization therapy. Besides MMP‐2, MMP‐3, and MMP‐9, other MMPs such as MMP‐1, MMP‐7, MMP‐8, and MMP‐12 have also been implicated in cerebral ischemia–reperfusion injury, although their specific roles and mechanisms are still being investigated.^46^, ^47^ Understanding the sources and effects of MMPs in the context of ischemic stroke provides insights into potential therapeutic strategies aimed at modulating their activity and minimizing the associated risks. In recent years, clinical evidence has increasingly supported the adverse impact of MMP‐9 in stroke patients undergoing thrombolysis therapy. The levels of MMP‐9 in plasma, both at baseline and at 3 hours post‐treatment, have been identified as potent predictors of cerebral parenchymal hemorrhages following administration. Additionally, MMP‐9 level serves as a potential biomarker for the continuous presence of cerebral ischemia.^48^, ^49^, ^50^ Moreover, exploratory clinical trials focusing on ischemic stroke have demonstrated that inhibition of MMP‐9 markedly reduces the breakdown of the BBB and neural tissue damage. This finding distinctly highlights the therapeutic potential of MMP‐9 inhibition in the treatment of ischemic stroke.^51^ Looking ahead, there is substantial promise in the development and application of anti‐MMP drugs. These drugs may potentially be combined with standard ischemia–reperfusion therapies to mitigate hemorrhagic complications and enhance overall stroke outcomes.

## 4 CONVERGENT REGULATION OF BLOOD–BRAIN BARRIER INTEGRITY BY INTERLINKED GSK‐3Β SIGNALING CASCADES

The BBB is a specialized protective barrier that regulates transport between the circulatory system and the central nervous system. It is formed by brain microvascular endothelial cells, pericytes, astrocytic end‐feet processes, and basement membrane components.^52^ Tight junctions between endothelial cells limit paracellular diffusion, maintaining a highly selective permeability barrier. The integrity of the BBB is dependent on intricate signaling mechanisms that regulate endothelial tight junction assembly, vascular permeability, and neuroinflammation.

Among the pathways involved after acute ischemic stroke, three key regulators of blood–brain barrier function are the Wnt/β‐catenin, Notch/Dll4, and Unc5B/Netrin‐1 signaling cascades. These evolutionarily conserved pathways are fundamentally interlinked at the level of downstream effector molecules to promote BBB stability through convergent mechanisms. Canonical Wnt signaling inhibits the destruction complex to allow β‐catenin accumulation and nuclear translocation, thereby regulating gene expression related to endothelial quiescence and intercellular adhesion.^53^ The Notch pathway similarly prevents proteasomal degradation of its intracellular domain upon ligand binding, permitting transcriptional regulation of tight junction proteins.^54^ Activation of the Unc5B dependence receptor by Netrin‐1 triggers downstream signaling via PI3K/Akt to endothelial survival factors and Wnt/β‐catenin enhancement.^55^

**FIGURE 3:**
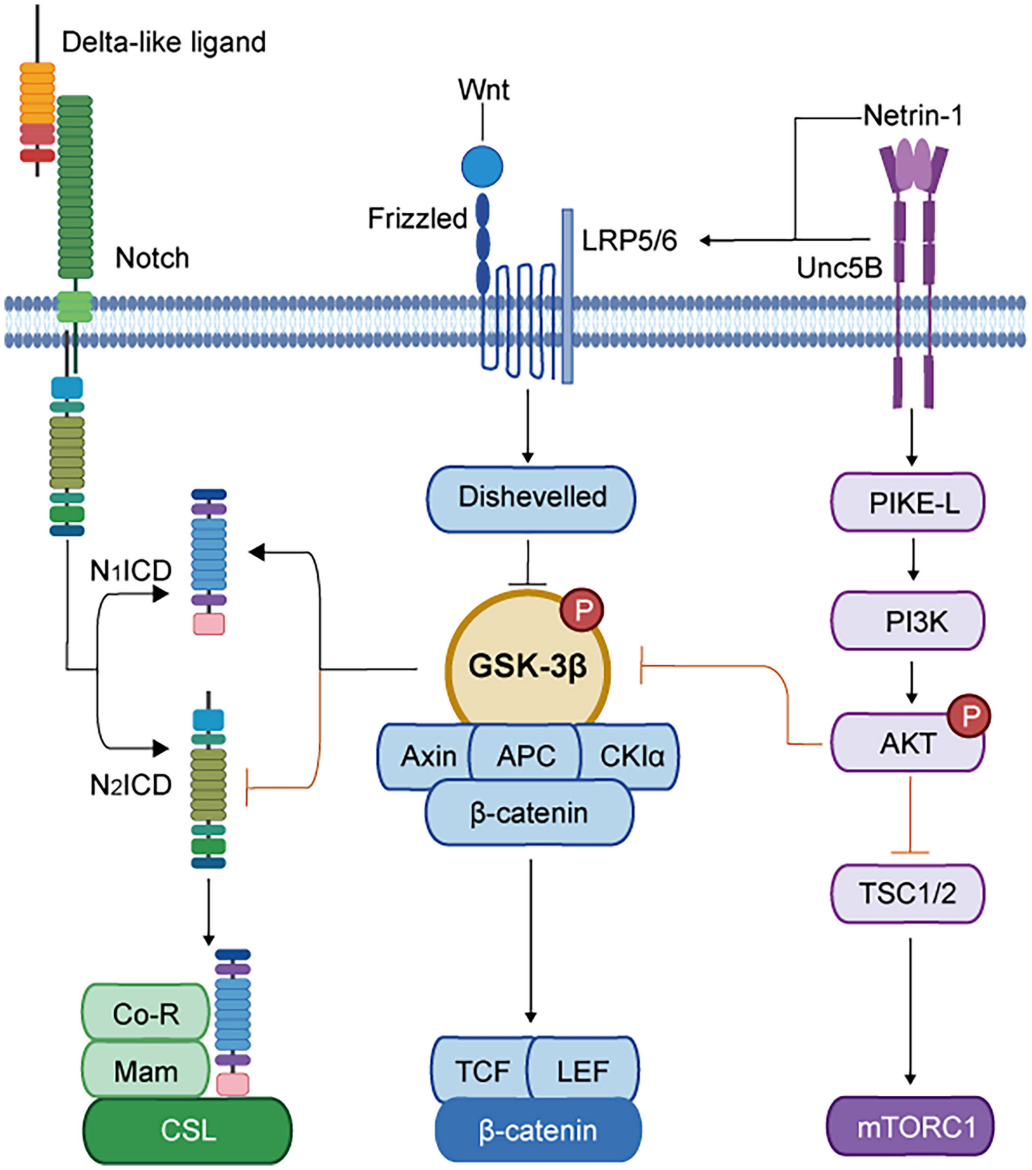
GSK‐3β as a converging point in key signaling pathways regulating BBB integrity post‐stroke. GSK‐3β emerges as a pivotal molecular hub in the intricate network of signaling pathways regulating the BBB following stroke. GSK‐3β's multifaceted role is depicted through its interaction with the Wnt signaling pathway, the Notch/Dll4 pathway, and the Unc5B/Netrin‐1 signaling pathway. Key molecules of each signaling pathway form corresponding transcription complexes to regulate gene expression. APC, adenomatous polyposis coli; CSL, DNA‐binding protein; GSK‐3β, glycogen synthase kinase‐3β; LRP5/6, lipoprotein receptor‐related proteins 5 and 6; Mam, CSL's co‐activator Mastermind; PI3K, phosphatidylinositol3‐kinaseNICD, notch intracellular domain; TCF/LEF, T‐cell factor/lymphoid enhancer factor. Formation of corresponding transcription complexes to regulate gene expression.

Though initiated by distinct ligands, these three signaling systems share the key intracellular mediator glycogen synthase kinase‐3β (GSK‐3β). By modulating its protein stability, they exert overlapping protective effects on cerebrovascular integrity after acute ischemic stroke. Pharmacological agents targeting shared downstream molecules like GSK‐3β to stabilize β‐catenin hold therapeutic promise for acute ischemic stroke (Figure 3). However, timing and dosage must be optimized to leverage the vasoprotective effects while avoiding potential side effects. Elucidating the coordinated activity of these fundamental signaling networks will provide insight into molecular strategies for preserving BBB function after cerebral ischemia–reperfusion injury.

### 4.1 Wnt signaling pathway

The Wnt signaling pathway is a complex network of proteins best known for their roles in embryogenesis and cancer, but also involved in normal physiological processes in adult animals.^56^, ^57^ Wnt proteins bind to receptors of the Frizzled family and several co‐receptors such as low‐density lipoprotein receptor‐related proteins 5 and 6 (LRP5 and LRP6), Ryk, or Ror2.^58^, ^59^ This binding triggers the activation of the intracellular protein disheveled, which inhibits the destruction complex composed of the proteins Axin, Adenomatous polyposis coli (APC), and GSK3.^57^ Under normal conditions, this destruction complex promotes the degradation of β‐catenin, a key effector in the Wnt signaling pathway. Upon Wnt binding to its receptors, the destruction complex is inhibited, leading to the accumulation of β‐catenin in the cytoplasm. The stabilized β‐catenin then translocates into the nucleus where it interacts with T‐cell factor/lymphoid enhancer factor (TCF/LEF) family transcription factors to regulate the expression of Wnt target genes.^56^, ^57^ In addition to this canonical Wnt/β‐catenin signaling pathway, there are also non‐canonical Wnt signaling pathways that operate independently of β‐catenin. These include the Wnt/planar cell polarity pathway and the Wnt/Ca^2+^ pathway.^60^ The regulation of Wnt signaling is complex and involves a delicate balance between canonical and non‐canonical pathways, mis‐regulation of which can lead to developmental defects or diseases in adults.

Wnt signaling plays a crucial role in the regulation of many processes including cell fate determination, cell migration, neural patterning, and organogenesis during embryonic development.^61^ In adults, it contributes to homeostasis and regeneration of tissues such as the skin, blood, and lining of the intestine. In the context of cerebral ischemia, both activation and inhibition of Wnt signaling pathway have been shown to play a multifaceted role in regulating BBB integrity and vascular function. Recent research progress has highlighted the potential therapeutic value of targeting this pathway in stroke treatment and studies have shown that the Wnt/β‐catenin signaling pathway plays a crucial role in maintaining the integrity of the BBB and its function during cerebral ischemic stroke.^62^ For instance, astrocytes lacking pH‐sensitive NHE1 protein were found to induce Wnt production, promoting BBB repair after ischemic stroke.^63^ Similarly, transplantation of oligodendrocyte precursor cells was shown to protect the BBB in the acute phase of ischemic stroke via activating the Wnt/β‐catenin pathway.^64^ Another study found that JLX001, a novel compound, attenuated BBB dysfunction in rats with MCAO/R via activating the Wnt/β‐catenin signaling pathway.^65^ Inhibition of the immunoproteasome LMP2 ameliorated ischemia/hypoxia‐induced BBB injury through the Wnt/β‐catenin signaling pathway.^66^ Interestingly, normalization of non‐canonical Wnt signaling does not compromise BBB protection conferred by upregulating endothelial Wnt/β‐catenin signaling following ischemic stroke.^67^ This suggests that a balance between canonical and non‐canonical Wnt signaling pathways may be crucial for maintaining BBB integrity.

These findings highlight the complex role of the Wnt/β‐catenin signaling pathway in regulating BBB function during cerebral ischemia. The timing and extent of activation or inhibition of this pathway could significantly influence outcomes following ischemic stroke. Therefore, therapeutic strategies targeting this pathway could potentially improve recovery after stroke. However, further research is needed to fully understand these mechanisms and develop effective treatments.

### 4.2 The Notch/Delta‐like ligand 4 (Dll4) pathway

Similar to Wnt signaling pathway, the Notch/Dll4 pathway is a highly conserved cell signaling system that plays a fundamental role in various biological processes, including the regulation of blood vessel morphogenesis and remodeling.^68^, ^69^ The Notch signaling pathway is initiated when the Dll4, a single‐pass transmembrane protein predominantly expressed by the vascular endothelium, binds to one of the four Notch receptors (NOTCH1, NOTCH2, NOTCH3, and NOTCH4) on a neighboring cell and release the Notch intracellular domain (NICD).^69^ NICD inhibits β‐catenin activity directly by forming a complex that prevents β‐catenin binding its target sites and instead recruits β‐catenin to downstream target sites of Notch.^70^ However, the outcome of this interaction varies as N_1_ICD is positively regulated, while the activity of N_2_ICD is negatively regulated by GSK‐3β, depending on the upstream ligand.

The Notch/Dll4 pathway is known to regulate endothelial cell–cell interactions and tight junction assembly, both of which are crucial for maintaining the integrity of the BBB.^68^ Dysregulation of this pathway can disrupt these interactions, further compromising BBB integrity. For instance, it was found that treatment with Dl‐NBP, an FDA‐approved agents for ischemic stroke, was shown to promote functional recovery after ischemic stroke.^71^ This recovery was associated with upregulated white matter integrity, microvessels, and the tight junction protein occludin. Importantly, Dl‐NBP also promoted the expression of hypoxia‐induced factor‐1α (HIF‐1α), vascular endothelial growth factor (VEGF), Notch, and Dll4. These findings suggest that Dl‐NBP treatment could promote functional recovery after focal transient ischemia stroke by modulating the Notch/Dll4 pathway and highlights the potential of targeting this pathway as a therapeutic strategy in the acute phase after stroke. However, further research is needed to fully understand the mechanisms underlying the effects of Dl‐NBP on the Notch/Dll4 pathway to consider potential side effects as well.

### 4.3 The Unc5B/Netrin‐1 signaling pathway

The Unc5B/Netrin‐1 signaling pathway is a crucial regulator of angiogenesis and neuronal development, and it has been implicated in modulating the phosphatidylinositol3‐kinase (PI3K)/Akt/mTOR pathway.^72^, ^73^, ^74^, ^75^ The Unc5B receptor, a member of the dependence receptor family, can induce two opposite intracellular signaling cascades depending on the presence or absence of its ligand, Netrin‐1. In the presence of Netrin‐1, Unc5B promotes cell survival and is known to play an essential role in arbitrating blood vessel formation and axonal migration.^55^ Mechanistically, Netrin‐1 triggers the interaction of Unc5B with brain‐specific GTPase PIKE‐L, prompting the PI3K cascade which suppresses apoptosis. This interaction also influences endothelial cell survival and tight junction formation, which are critical for maintaining the integrity of the BBB.^55^ Inducible endothelial‐specific deletion of Unc5B in adult mice led to region and size‐selective BBB opening. Loss of Unc5B decreased BBB Wnt/β‐catenin signaling, and β‐catenin overexpression rescued Unc5B mutant BBB defects. Mechanistically, Netrin‐1 enhanced Unc5B interaction with the Wnt co‐receptor LRP6, induced its phosphorylation, and activated Wnt/β‐catenin downstream signaling.^55^

GSK‐3β is involved in multiple other signaling pathways, such as the insulin signaling pathway, the receptor tyrosine kinase, and mTOR pathways which involved in cell proliferation and apoptosis as well.^76^, ^77^, ^78^ Moreover, GSK‐3β can phosphorylate a variety of substrates other than those in the β‐catenin involved pathway, such as glycogen synthase and other metabolic enzymes, transcription factors CREB binding protein, c‐Myc and c‐Jun.^79^, ^80^ Collectively, GSK‐3β and its downstream β‐catenin appears to be a central regulator of BBB stability. Activation of multiple upstream signaling pathways, including the Notch/Dll4, the Unc5B/Netrin‐1, and finally the canonical Wnt, would lead to the TCF/ LEF‐based transcription complex assembly and transcription of related genes and finally maintain the function of BBB. These findings highlight the potential therapeutic use of GSK‐3β inhibitor, such as Lithium chloride (LiCl) for the purpose of BBB protection. This broad range of effects from GSK‐3β inhibition could lead to various side effects and include increased urination, shakiness of the hands, and increased thirst. Serious side effects include hypothyroidism, diabetes insipidus, and lithium toxicity. Therefore, while lithium chloride shows promise as a potential therapeutic agent for acute ischemic stroke, further research is needed to fully understand its mechanisms of action and develop strategies for its safe and effective use in clinical settings.

## 5 DISTINCT MOLECULAR SIGNALING EFFECTS ON BBB FUNCTION

Having discussed the coordinated control of BBB integrity by interlinked signaling cascades converging on shared mediators, there are also secreted factors which have been shown to modulate endothelial cell tight junctions, adhesion molecules, survival signaling, and permeability through their respective receptor‐mediated pathways. This section will summarize current knowledge on the effects of individual protein signaling molecules and their downstream cascades on BBB structure and function following cerebral ischemia–reperfusion injury. The timing and extent of signaling activation by secreted factors including platelet‐derived growth factor CC (PDGF‐CC), VEGF, and transforming growth factor β1 (TGF‐β1) can dramatically impact outcomes, with early transient effects often being detrimental due to exacerbated vascular leakage, while delayed and sustained signaling is generally protective for the BBB. In addition to directly influencing endothelial cells, these secretory molecules also modulate other cell types such as pericytes, astrocytes, microglia, and neurons to indirectly affect BBB integrity through multifaceted mechanisms. Elucidating the nuances of these distinct signaling pathways and cell‐type specific effects is key to understanding the dynamics of BBB disruption after ischemic stroke and developing targeted molecular therapies.

### 5.1 PDGF‐CC/PDGFRα signaling pathway

PDGF‐CC emerges as a key player affecting neurons and endothelial cells in the acute phase of ischemic stroke. For instance, in mouse stroke models, exogenous PDGF‐CC administration decreased infarct volume and improved functional outcomes through anti‐apoptotic signaling in neurons, which indicate the PDGF‐CC's direct neuroprotective role.^81^ However, PDGF‐CC may also negatively impact blood–brain barrier integrity. In a mouse model of stroke, activation of PDGF‐CC attenuated BBB leakage, suggesting it may promote increased vascular permeability after ischemia.^82^, ^83^ The interaction between PDGF‐CC and the thrombolytic agent tPA requires further investigation, as both are upregulated early after ischemic stroke.^83^, ^84^ Understanding the influence of PDGF‐CC and its association with rtPA substrate is crucial for comprehending the complex dynamics of blood–brain barrier integrity during the acute phase of ischemic stroke.

### 5.2 The VEGF signaling pathway

The VEGF signaling pathway plays a multifaceted role in regulating blood–brain barrier integrity and vascular function in the context of cerebral ischemia. In the acute injury phase, multiple studies have shown VEGF administration in animal stroke models increases vascular permeability, leading to vasogenic edema and hemorrhagic transformation.^85^, ^86^ Possible mechanisms include VEGF‐mediated dissociation of tight junction proteins like occludin and ZO‐1, increased activity of permeability factors like nitric oxide (NO) and MMPs, and enhanced vesicular transport across endothelial cells.^87^, ^88^ However, starting from 3 to 7 days post‐ischemia, VEGF promotes angiogenesis, neurogenesis and neurovascular remodeling necessary for tissue repair and functional recovery.^86^, ^89^ In the chronic phase, VEGF stimulates endothelial progenitor cell mobilization and differentiation to regenerate damaged cerebrovasculature.^90^ It also exerts neurotrophic and neuroprotective effects, reducing neuronal apoptosis and enhancing neural plasticity.^91^, ^92^

Interestingly, studies have shown that diabetes can exacerbate the up‐regulation of VEGF, leading to aggravated hemorrhage after experimental cerebral ischemia and delayed reperfusion.^93^ This suggests that conditions such as diabetes can modulate the effects of VEGF on BBB integrity and vascular function. Moreover, early post‐stroke activation of VEGF receptor 2 has been found to hinder the receptor 1‐dependent neuroprotection afforded by the endogenous ligand, which highlights the importance of not only the timing but also the specific receptor‐mediated actions of VEGF in determining its effects on stroke outcomes.^94^, ^95^

The timing and amount of VEGF delivery is critical, as excess levels in the acute stage can worsen injury, while controlled expression during recovery promotes regeneration. Short‐term anti‐VEGF therapy in the initial injury phase, followed by pro‐angiogenic VEGF treatment in the late phase, could represent an optimal therapeutic approach.^96^ Overall, the biphasic responses of CNS cells to VEGF signaling underscore its multi‐modal effects on vascular integrity, neural viability, and brain repair after ischemic stroke. Careful consideration must be given to the timing and dosage of VEGF‐targeted therapies. Short‐term anti‐VEGF therapy in the initial injury phase, followed by pro‐angiogenic VEGF treatment in the late phase, could represent an optimal therapeutic approach.

### 5.3 The TGF‐β1 signaling pathway

In addition to PDGF‐CC and the VEGF signaling pathway, various other factors are implicated in the protection and repair of blood–brain barrier function. TGF‐β1, a multifunctional cytokine, emerges as a potential candidate, as it regulates tight junction proteins, modulates inflammatory responses, and promotes endothelial cell survival.

TGF‐β1 signaling is critical for maintaining BBB integrity by regulating tight junction assembly between endothelial cells. TGF‐β1 treatment reduced blood–brain barrier permeability following ischemic stroke in mice and rats by upregulating expression of tight junction proteins like occludin, claudin‐5, and ZO‐1.^97^, ^98^ Conversely, disruption of TGF‐β1 signaling via knockout of downstream Smad proteins or TGF‐β receptor inhibition exacerbated BBB leakage after stoke modeling.^99^ Mechanistically, TGF‐β1 promotes tight junction integrity and strength by activating signaling pathways like PI3K/Akt and MEK/ERK.^100^ It also exerts anti‐inflammatory effects on resident microglia and infiltrating leukocytes, attenuating production of permeability‐inducing factors like inflammatory cytokines, ROS, NO, and MMPs.^101^, ^102^ Delayed and sustained TGF‐β1 delivery appears optimal, as early transient administration can worsen injury by promoting NLRP3 inflammasome activation in microglia.^103^

In addition to tight junction regulation, TGF‐β1 signaling also promotes pericyte differentiation and coverage of endothelial cells, further reinforcing the neurovascular unit.^104^ Studies utilizing 3D BBB models suggest TGF‐β1's protective effects may involve differentiated pericytes secreting factors that stabilize endothelial tight junctions.^105^ Overall, TGF‐β1 signaling intricately regulates multiple cell types to maintain the delicate balance of BBB permeability after ischemic insult.

### 5.4 The SDF‐1α/CXCR4 signaling pathway

The SDF‐1α/CXCR4 pathway is a key signaling axis influencing BBB integrity and vascular function after ischemic stroke. SDF‐1α, often referred to as CXCL12, is a chemokine that activates downstream signaling pathways by binding to the receptor CXCR4 on endothelial cells. Studies in rodent models have shown exogenous SDF‐1α treatment within 24 h after induced stroke reduces BBB leakage and hemorrhagic transformation.^106^, ^107^ Mechanistically, the binding of SDF‐1α to CXCR4 stimulates several downstream cascades involved in tight junction regulation, including PI3K/Akt, ERK1/2, JAK2/STAT3, and RhoA/Rock pathways.^108^ Through these signaling systems, SDF‐1α stabilizes and promotes expression of tight junction proteins claudin‐5, occludin, and ZO‐1 in cerebrovascular endothelial cells after ischemic injury. In addition to preserving tight junctions, SDF‐1α/CXCR4 signaling also stimulates angiogenesis and new blood vessel formation via VEGF/eNOS pathways to revascularize the neurovascular unit after stroke.^109^

The effects of SDF‐1α/CXCR4 are time‐sensitive, as delayed treatment several days after initial stroke appears to optimize functional improvements, suggesting early manipulation may interfere with necessary inflammatory processes.^109^ Overall, leveraging the multifaceted neurovascular protective effects of endogenous SDF‐1α/CXCR4 signaling presents a promising avenue for developing stroke therapies to maintain BBB integrity after ischemia.

In summary, the effect of secreted protein molecules and their downstream signals on the integrity of the blood–brain barrier in ischemic stroke is a complex and dynamic process. Analyzing the distinct effects and mechanisms of pathways like PDGF‐CC, VEGF, TGF‐β1, and SDF‐1α/CXCR4 highlights their multifaceted roles in maintaining BBB structure and function after injury. Further research on leveraging these endogenous signaling systems could aid the development of therapies to preserve cerebral vascular integrity following ischemic stroke.

## 6 ENDOTHELIAL CELL DEATH PATHWAYS

**FIGURE 4:**
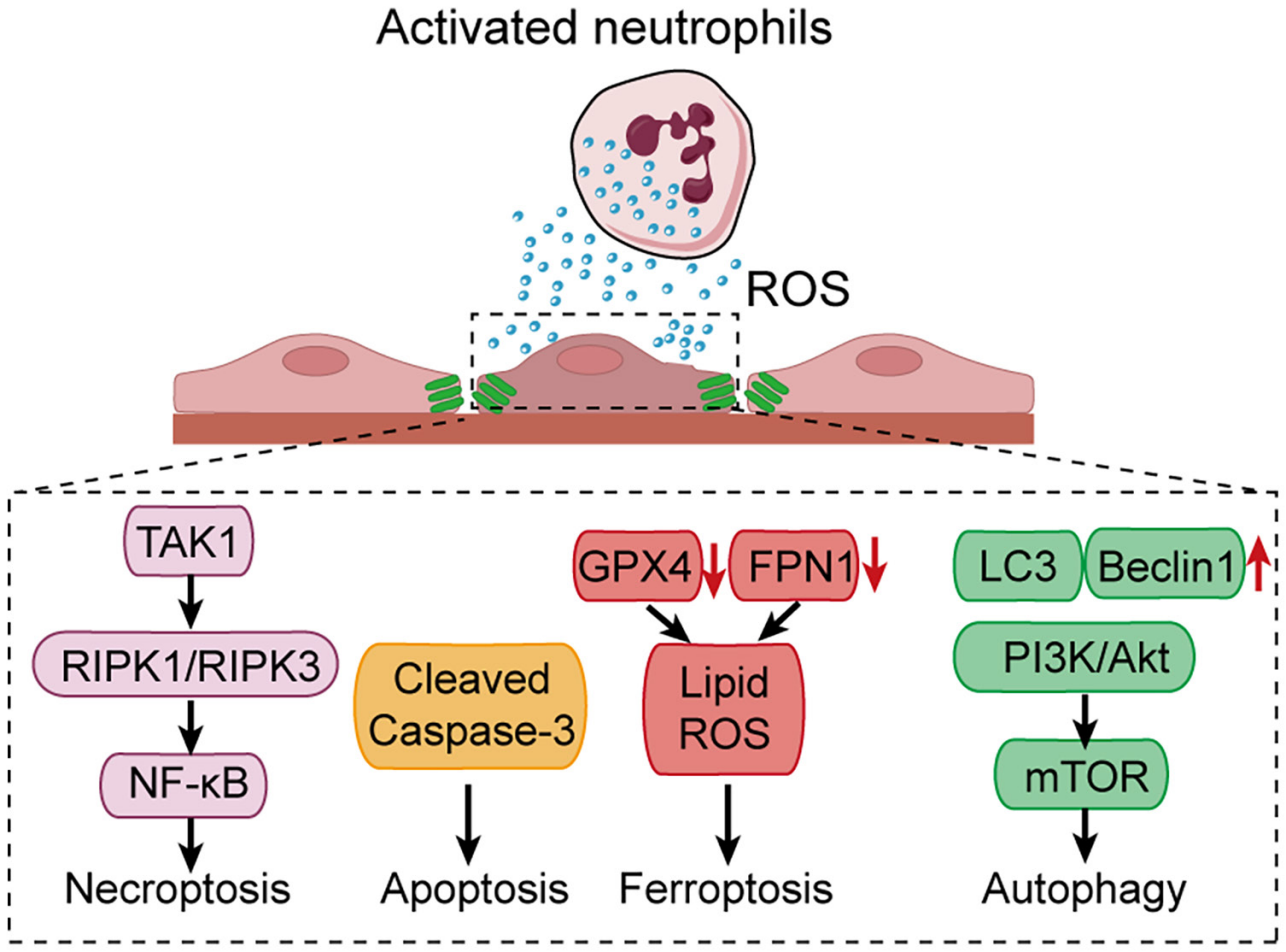
Programmed endothelial cell death pathways. Cerebrovascular endothelial cell death after ischemia–reperfusion injury can occur through several programmed modalities, including apoptosis, necroptosis, ferroptosis, and autophagy, which ultimately leads to disruption of the blood–brain barrier.

Cerebrovascular endothelial cell death after ischemia–reperfusion injury can occur through several programmed modalities, including apoptosis, necroptosis, ferroptosis, and autophagy (Figure 4). Apoptosis features cell shrinking, chromatin condensation, and DNA fragmentation.^110^ Necroptosis, also known as programmed necrosis, is featured with cell swelling and membrane rupture. Ferroptosis is characterized by lethal lipid peroxidation.^111^ Autophagy is a process of cellular self‐digestion, which features the formation of double‐membrane autophagosomes that fuse with lysosomes to degrade cell contents. The causes of programmed cell death in cerebrovascular endothelial cells play a crucial role in understanding the side effects associated with vascular recanalization therapies, such as hemorrhagic transformation and intracerebral hemorrhage. This section provides an overview of different types of programmed cell death, including apoptosis, necroptosis, ferroptosis, and autophagy in the context of ischemia–reperfusion injury.^112^

### 6.1 Apoptosis, necroptosis, ferroptosis, and autophagy after ischemia–reperfusion

Apoptosis, the programmed cell death, is a highly regulated cell death program characterized by nuclear condensation, DNA fragmentation, cell shrinkage, membrane blebbing, and the formation of apoptotic bodies. Apoptosis is induced by caspases, which are cysteine proteases responsible for cleaving important cellular components. This cellular process can be initiated either by the extrinsic death receptor pathway or the intrinsic mitochondrial pathway, both ultimately leading to the activation of executioner caspases. Dysregulation of apoptotic signaling and oxidative stress‐induced vascular injury and integrity disruption play a pivotal role in the initiation and development of IS and contribute to neuronal cell death and BBB disruption after ischemic stroke and recanalization therapy.^113^ Excessive endothelial apoptosis is implicated in worse outcomes and hemorrhagic transformation following thrombolysis In MCAO mice, miR‐503 level was significantly increased in brain and contributed to endothelial cell (EC) dysfunction by promoting apoptosis. While inhibition of miR‐503 ameliorated apoptosis of ECs, oxidative stress, BBB permeability, and neurological damage after stroke.^114^

Necroptosis, also termed programmed necrosis, is triggered by binding of agonistic ligands such as TNF‐α, FasL, and TRAIL to their respective death receptors. A key feature of necroptosis is that it predominates when apoptotic signaling is inhibited or deficient, representing an alternative cell death pathway. In the absence of apoptotic caspase activation, necroptotic signaling cascades mediated by RIPK1/RIPK3 propagate and execute regulated necrotic cell death. Understanding the molecular switch between apoptotic and necroptotic signaling may reveal therapeutic strategies to modulate cell death mechanisms after ischemia–reperfusion injury. The activation of RIPK1 in cerebrovascular ECs is one of the primary deleterious mechanisms in the brain cell death and is critical for mediating apoptosis and necroptosis following ischemic insult. The downstream consequences of RIPK1 activation exhibit cell‐type specificity: the activation of RIPK1 in endothelial cells promotes necroptosis and cerebrovascular damage which mediate intracerebral hemorrhage, the activation of RIPK1 in neurons promotes both necroptosis and apoptosis in a time‐delayed fashion. Furthermore, inhibition of RIPK1 kinase can reduce the stroke infarction volume.^115^

Ferroptosis is a form of regulated cell death characterized by the iron‐dependent accumulation of lethal lipid ROS, closely linked to the occurrence and development of stroke, and a common pathway of cell death in ischemic stroke. It is morphologically, biochemically, and genetically distinct from other cell death modalities. A key trigger of ferroptosis is the inhibition of glutathione peroxidase 4 (GPX4), an enzyme that protects cells from lipid peroxidation. When GPX4 is inactivated, lipid ROS accumulate to toxic levels, inducing oxidative damage to lipids, proteins, DNA and ultimately driving ferroptotic cell death. Ferroportin 1 (FPN1) is the only known cellular iron export protein in cerebral endothelial cells. *Fpn1* knockout in ECs attenuated oxidative stress and inflammatory responses after stroke and inhibited ferroptosis and apoptosis, ultimately attenuating neurological damage and reducing cerebral infarct volume during acute phase ischemic attacks.^116^Understanding mechanisms of ferroptosis regulation has implications for mitigating tissue damage in contexts like ischemic injury, where iron accumulation and lipid peroxidation are key factors. Further research into modulating ferroptotic signaling may reveal therapeutic strategies after ischemia–reperfusion.

Autophagy is a highly regulated cellular degradation process featuring the formation of double‐membrane autophagosomes that engulf cytoplasmic material and fuse with lysosomes for breakdown. It plays dual roles in cerebral ischemia, with moderate induction initially protecting cells by removing damaged components, but excessive autophagy contributing to autophagic cell death. Key mediators of autophagy signaling include Beclin‐1, which interacts with Bcl‐2 family proteins to regulate autophagosome formation. Activation of the autophagy‐lysosomal pathway after ischemia normalized the permeability of BBB, while the inhibition of autophagy prevents damage to brain microvascular endothelial cells after reperfusion.^117^

### 6.2 Key factors contributing to endothelial programmed cell death

Key factors contributing to endothelial programmed cell death after ischemia–reperfusion include oxidative stress, mitochondrial dysfunction, and calcium overload.^118^, ^119^ Excessive ROS are mainly generated from the mitochondrial electron transport chain, xanthine oxidase, NADPH oxidases, and activated neutrophils.^120^ Ischemia depletes cellular antioxidants, and abrupt reoxygenation upon reperfusion further drives ROS production beyond the cell's scavenging capacity. This oxidative damage causes lipid peroxidation, protein misfolding, DNA breakage, and mitochondrial impairment, ultimately inducing signaling cascades leading to apoptosis, necroptosis, autophagy, and ferroptosis. Therapeutic strategies aimed at suppressing ROS generation or enhancing antioxidant capacity may mitigate oxidative stress‐induced endothelial cell death. For example, inhibition of S1PR3 aggravated H_2_O_2_‐induced endothelial hyperpermeability and tight junction disruption in bEnd.3 cell monolayer, while S1PR2 blockade ameliorated oxidative stress‐induced cerebrovascular endothelial hyperpermeability.^121^ Leonurine provides neuroprotection through the NO/nitric oxide synthase (NOS)‐mediated oxidative stress signaling pathway.^122^ These studies may help us gain a new insight into exploring potential drug targets for oxidative stress‐induced cerebrovascular disease.

Mitochondrial damage is both a consequence of and contributor to endothelial cell death after ischemia–reperfusion. Hypoxic damage to mitochondria generates ROS upon reperfusion and causes release of pro‐apoptotic factors like cytochrome c. Permeability transition pore opening, loss of membrane potential, and impaired oxidative phosphorylation compromise cellular energetics. Meanwhile, dysfunctional mitochondria release calcium stores, disrupting calcium homeostasis, which further promotes endothelial cell death through both necrosis and apoptosis. Ischemia causes ATP depletion, leading to failure of ATP‐dependent calcium pumps and accumulation of intracellular calcium. Mitochondrial damage and oxidative stress further impair calcium buffering. Excessive calcium activates phospholipases, proteases, and endonucleases that degrade cellular components. Calcium overload also triggers the mitochondrial permeability transition and release of pro‐apoptotic factors. Restoring calcium regulation through calcium channel inhibition or anti‐oxidant treatment could potentially mitigate endothelial cell death after cerebral ischemia–reperfusion.

### 6.3 Interactions and interconversions between death modalities

Not only do the programmed cell death overlap in time of occurrence but also may undergo interaction or conversion from one to another in the setting of ischemia–reperfusion injury.^115^ For example, caspase‐8, a key mediator of apoptosis, can also inhibit necroptosis by cleaving and inactivating RIPK1 and RIPK3 necrosome proteins. When caspase‐8 is inhibited, necroptosis is promoted instead of apoptosis.^123^ Meanwhile, calpain released from lysosomes during autophagy can promote apoptosis through Bid and Bcl‐2 cleavage. The other example is active caspase‐3 which can cleave and inactivate PARP‐1 and block its necrosis‐inducing effects. On the other hand, PARP‐1 hyperactivation can deplete cellular energy stores and induce AIF translocation to the nucleus, switching the cell towards regulated necrosis.^124^ The balance of activity of these factors can determine if a cell undergoes apoptosis versus necroptosis.

Autophagy may suppress necrosis by removing damaged mitochondria, but excessive autophagy can also facilitate necrosis in apoptosis‐deficient cells. In addition, selective autophagy of factors like ferritin may drive ferroptosis by promoting iron accumulation and lipid peroxidation. Overall, the interplay between autophagy, apoptosis, necroptosis, and ferroptosis involves multifaceted modulatory effects that require further elucidation in the setting of cerebral ischemia–reperfusion injury. Understanding these interconnections may reveal ways to modulate cell death mechanisms therapeutically after ischemia–reperfusion injury.

In summary, the very early stage of cerebral ischemia–reperfusion injury is characterized by increased ROS generation, which promotes programmed cell death in cerebrovascular endothelial cells. Mitochondrial dysfunction and overactivated neutrophils are the main sources of ROS during this critical period. Understanding the role of these sources in the generation of ROS provides valuable insights into the mechanisms underlying programmed cell death and offers potential targets for therapeutic interventions to mitigate the adverse effects of cerebral ischemia–reperfusion injury.

## 7 CONCLUSION

This review has synthesized current knowledge on the cellular and molecular mechanisms underlying acute cerebral ischemia–reperfusion injury in the early period following ischemic stroke. The complex interplay between endothelial cell damage, blood–brain barrier disruption, programmed cell death initiation, and deleterious inflammation and oxidative stress highlights the multifaceted nature of the early reperfusion injury process. Despite years of extensive research and significant advances in our understanding of stroke pathophysiology, the therapeutic options available for acute stroke continue to be remarkably limited. Though vascular recanalization therapies have shown efficacy in treating ischemic stroke, further research is needed to improve outcomes and mitigate complications like hemorrhagic transformation. Elucidating the nuances of neutrophil‐mediated cerebrovascular injury, MMP release dynamics, protein signaling involved in BBB integrity, and endothelial cell death pathways will reveal potential targets for adjunctive neuroprotective agents. Identifying optimal therapeutic windows, drug delivery methods, and combinatorial approaches also warrants further investigation.

At present, various neuroprotective treatment strategies proposed for stroke, which focus on inhibiting the activity of MMPs, have demonstrated neuroprotective effects in the early stages following an ischemic stroke. Neuroprotective approaches that target specific signaling pathways involved in the pathogenesis of ischemic stroke have also shown promise in animal experiments. However, there remains a notable lack of clinical evidence regarding the efficacy of these therapeutic strategies in human patients. Only a few neuroprotective agents, such as minocycline and edaravone, have progressed to clinical trials. Consequently, further clinical research is imperative to refine these strategies for potential neuroprotection. This research should aim to determine whether a single neuroprotectant or a combination of agents targeting different neuroprotective pathways is effective in treating stroke patients. Such studies are crucial for advancing the basic understanding and clinical application of effective neuroprotective strategies.

## AUTHOR CONTRIBUTIONS

QH and YW designed and wrote the manuscript. CF, ZF, MY, JH revised the manuscript. YM and ZM gave constructive advice and participated in proofreading of this paper. All authors contributed to the article and approved the submitted version.

## FUNDING INFORMATION

This work was supported by the National Natural Science Foundation of China (82204983 to Z.M., 82273923 to Y.M.), Shenzhen Science and Technology Program (JCYJ20220531092010023 to Z.M., JCYJ20220531100203008 to Y.M., JCYJ20230807140721043 to C.F.).

## CONFLICT OF INTEREST STATEMENT

All authors claim that there are no conflicts of interest.

## Data Availability

Data sharing is not applicable to this article as no new data were created or analyzed in this study.
